# Fault Injection Emulation for Systems in FPGAs: Tools, Techniques and Methodology, a Tutorial

**DOI:** 10.3390/s21041392

**Published:** 2021-02-17

**Authors:** Óscar Ruano, Francisco García-Herrero, Luis Alberto Aranda, Alfonso Sánchez-Macián, Laura Rodriguez, Juan Antonio Maestro

**Affiliations:** ARIES Research Center, Universidad Antonio Nebrija, 28040 Madrid, Spain; oruano@nebrija.es (Ó.R.); laranda@nebrija.es (L.A.A.); asanchep@nebrija.es (A.S.-M.); lrodriguezs5@alumnos.nebrija.es (L.R.); jmaestro@nebrija.es (J.A.M.)

**Keywords:** communication modules, emulation, fault injection debugger, FIR filter, SEM IP, SEU, Xilinx

## Abstract

Communication systems that work in jeopardized environments such as space are affected by soft errors that can cause malfunctions in the behavior of the circuits such as, for example, single event upsets (SEUs) or multiple bit upsets (MBUs). In order to avoid this erroneous functioning, this kind of systems are usually protected using redundant logic such as triple modular redundancy (TMR) or error correction codes (ECCs). After the implementation of the protected modules, the communication modules must be tested to assess the achieved reliability. These tests could be driven into accelerator facilities through ionization processes or they can be performed using fault injection tools based on software simulation such as the SEUs simulation tool (SST), or based on field-programmable gate array (FPGA) emulation like the one described in this work. In this paper, a tutorial for the setup of a fault injection emulation platform based on the Xilinx soft error mitigation (SEM) intellectual property (IP) controller is depicted step by step, showing a complete cycle. To illustrate this procedure, an online repository with a complete project and a step-by-step guide is provided, using as device under test a classical communication component such as a finite impulse response (FIR) filter. Finally, the integration of the automatic configuration memory error-injection (ACME) tool to speed up the fault injection process is explained in detail at the end of the paper.

## 1. Introduction

The reliability feature for communication systems that must work in harsh environments such as space or radioactively contaminated areas, is a major concern nowadays [1,2]. For these scenarios, not only area, delay and power consumption play an important role in the design process, but also fault tolerance is mandatory in order to deal with soft errors such as single event upsets (SEUs), multiple bit upsets (MBUs) or single event functional interrupt (SEFIs) produced by radiation [3]. These upsets are caused by ionizing radiation strikes that alter the charge in storage elements such as configuration memory cells, user memory or registers, causing non-permanent errors in the systems. In order to avoid these errors, there are two main approaches.

The first option called radiation-hardening by process (RHBP), includes physical techniques that must change the manufacturing process by means of shielding or applying silicon on insulator (SOI) [4,5]. Nowadays, rad-tolerant components are expensive and some generations older than the no rad-hard technology, which has led to the emergence of alternatives such as the radiation-hardening by design approach (RHBD) [6].

In RHBD approaches, the manufacturing processes are not modified to meet a specified radiation constraint. The techniques employed to meet these requirements are implemented in the VLSI architecture instead. This methodology applies some well-known hardening techniques based on spatial redundancy—e.g., triple modular redundancy (TMR) [7], information redundancy, error correction codes (ECC), or the “system knowledge” [8,9,10]—to increase the reliability of the design.

When the reliability parameter is introduced in the design workflow, it is important not only to design the protection schemes without penalizing hardware performance (area, timing and power), but also to be able to prove that the protected designs achieve the desired percentage of success under critical scenarios. To test the fault tolerance of protected designs, specific mechanisms and platforms are usually required. Fault injection is a feasible practice to achieve this purpose [11] by means of a platform capable of generating bit flips into the memory elements to emulate SEUs or MBUs. Fault injection platforms can be classified as [12]:

- Based on hardware fault injection.
- Based on software fault injection.
- Based on simulation fault injection.
- Based on emulation fault injection.

This work is focused on explaining in-depth the whole workflow of an emulation-based fault injection platform for SRAM-based field-programmable gate arrays (FPGAs) based on the Xilinx soft error mitigation (SEM) intellectual property (IP) core. As an example to illustrate the step-by-step explanation of the fault injection platform, the reliability of a widely used finite impulse response (FIR) filter will be studied. The examples provided in this work are available as an online resource in a repository to facilitate the understanding and replication of the results. Also, in order to improve this workflow, the use and integration of the automatic configuration memory error-injection tool (ACME) [13] is introduced. This tool and the whole framework described here, despite others such as the Fault Injection Intel^®^ FPGA IP Core [14], are totally open and free to be used by designers, providing the same amount of information and accuracy without requiring to purchase of a separate license.

This paper is organized as follows: Section 2 presents an example of a common communication system that is exposed to radiation effects in satellites and, hence, it is interesting to be tested through fault injection. This module is the superheterodyne receiver that includes, for example, FIR filters and coordinate rotation digital computer (CORDIC) modules. This section also shows the effects of SEUs in the frequency domain. Section 3 briefly introduces some fault injection tools that can be found in the literature, classifying them in terms of the strategy followed to perform the injection: software, hardware, emulation, or simulation. Section 4 describes the effects of radiation over SRAM-based FPGAs explaining the impact on routing and logic. Section 5 reviews fault-tolerant techniques for FPGAs based on the reconfiguration features like scrubbing. Section 6 depicts a whole platform setup for the fault emulation based on Xilinx’s technology including the controllers and the interconnections required between modules. Section 7 describes, step by step, the workflow for emulation-based injection with all the details for configuration of the tools involved and the related hardware. In Section 8, a tool named ACME [13], developed in order to automate and accelerate the fault campaigns, is introduced. This tool looks for those regions where the target circuits are located inside the FPGA in a pinpointed way. It allows the designer to avoid injections at irrelevant FPGA frames, speeding up the experiments. Section 9 shows a simple way to automatize the whole fault injection process with Matlab and includes a reference to a public repository to replicate the described workflow with any digital design for FPGA. Finally, conclusions are presented in Section 10.

### Graphical Index of the Paper Structure


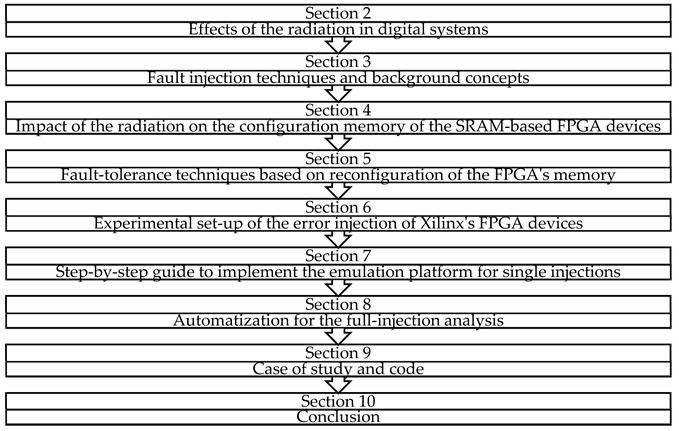


## 2. Reliability Assurance for Communication System Modules

**Figure 1:**
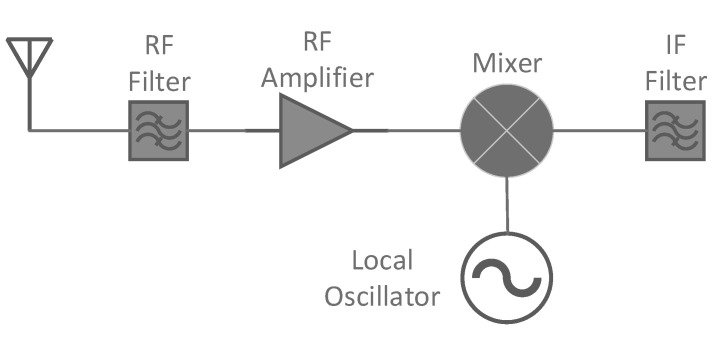
Basic components of a receiver.

Most of the communications system modules, such as equalizers, filters, mixers or demodulators, are widely used in radiation environments which can be affected by SEUs, as happens with satellite transceivers. One of the most classic examples that can be found in literature is the superheterodyne receiver (Figure 1). The digital signal processing (DSP) units implemented to process the received signals include different algorithms from FIR and infinite impulse response (IIR) filters, to direct digital synthesizers (DDS) obtained with CORDIC units and other modules, such as equalizers, to adjust the gain of the signal in different radio spectrum. All these modules, implemented with analog components in the past, are now designed and integrated in digital devices, first application-specific integrated circuits (ASICs) and now FPGAs. These modern architectures support reconfiguration when a change in the specifications is required. Also, FPGA devices allow a high integration and extensibility on the functionality of the communication modules. For example, new algorithms can be integrated with these devices without the requirement of changing the chips onboard, only by modifying the configuration file, i.e., changing the digital demodulators or the error correction codes applied for the transmissions.

As a first approach for space communications, space-grade rad-hard FPGAs were employed. However, their cost and reduced efficiency in terms of power and area, encouraged researchers to look for alternatives to protect the DSP units included in the different communication modules. One of these solutions is using an ad-hoc protection. The correct and efficient protection of these modules is totally critical since the effect of just one error (which in FPGAs is a permanent error, as we will see in the next sections of the paper) can totally modify the receiver operation mode and with this, the electromagnetic spectrum of the received signal.

As an example, in the following we will show the impact of one single error in the behavior of a digital receiver.

**Figure 2:**
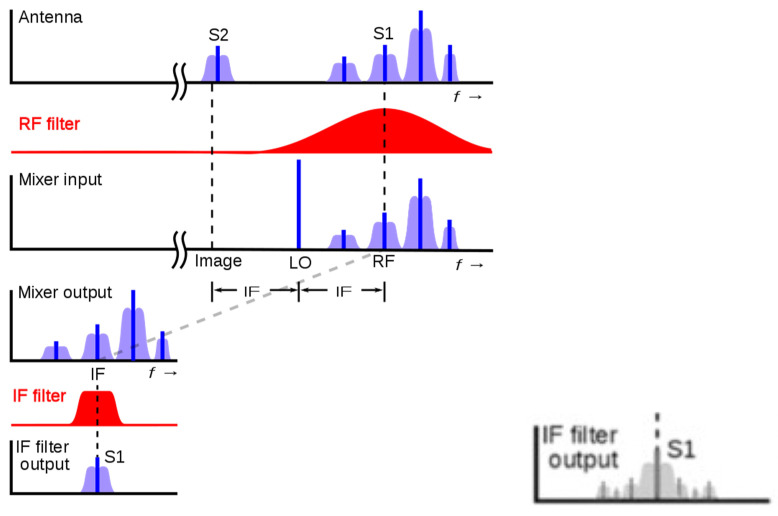
Operation mode in presence of an SEU (**right**) and without SEU (**left**).

Figure 2 shows how S1, which is the desired frequency, is processed with other incoming signals which need to be filtered like S2 (1st graph). These filtering processes are shown in both the second and fifth graphics. For example, the RF filter (2nd graph) removes any signal such as S2 at the image frequency local oscillator (LO)—intermediate frequency (IF). The remaining signal is applied to the mixer which implements a CORDIC processor where a sine or cosine wave with a frequency oscillator (3rd graph) is added.

At this point the signal S1 is combined with the LO frequency to create a heterodyne at the difference between these frequencies, the IF, at the mixer output (4th graph). Finally, it passes through the IF bandpass filter with and without an SEU (5th graph left and right respectively) and is amplified and demodulated.

**Figure 3:**
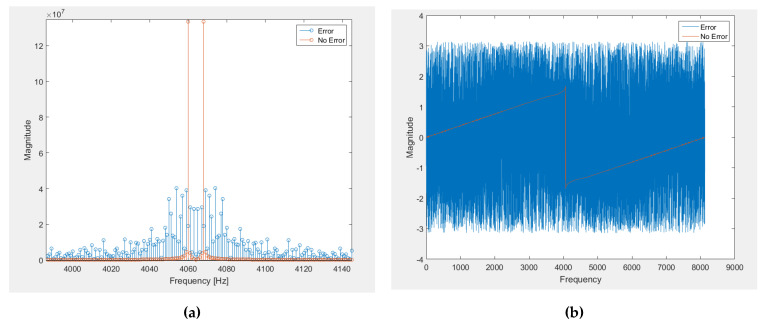
(**a**) Receiver’s Magnitude in presence of a SEU and (**b**) Receiver’s Phase presence in of a SEU.

As it can be noticed through this receiver example, the filtering processes are present in most of the stages of the communication module, so it is important to know what the impact of a single error in one filter is. In the next Figure 3, it can be seen the distortion that a tone suffers when it goes through a filter that has just one SEU (result of a real simulation). Comparing the output without the effect of radiation with the output with the radiation effects, the spectrum of the output signal is totally distorted including not only multiple harmonics but also a change in the phase, that will alter the received signal in a way that will make the message transmitted impossible to recover. The IF output of Figure 2, which just one error would be similar to Figure 3.

Hence, showing the great impact of just one error, it can be concluded that it is crucial to have an affordable and efficient tool and workflow that allows designers of space communication systems to analyze the effects of the faults caused by radiation.

The rest of the paper presents the fault injection emulation in depth. The workflow followed in this tutorial has been applied in many of these communication systems implemented in FPGA such as FIR, IIR, Bloom, Cuckoo and Parallels filters, that are available in the recent literature [15,16,17,18,19,20].

## 3. Background Concepts

**Figure 4:**
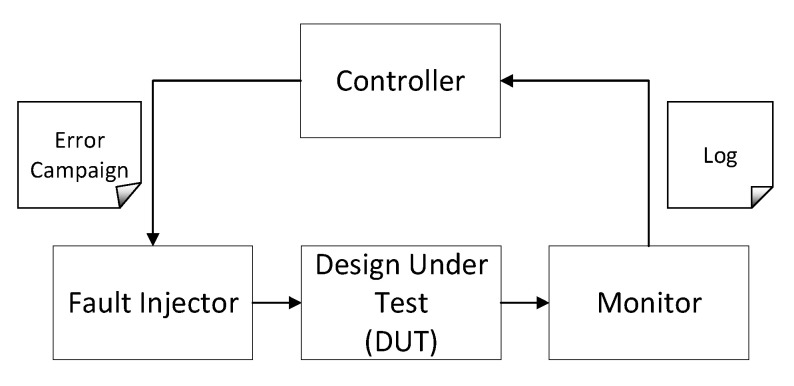
Basic components of an error injection environment.

Fault injection is a widely used technique for fault tolerance evaluation. Essentially, the fault injection methods presented in the literature have implemented hardware as well as software components. A common architecture for this kind of systems is presented in Figure 4.

where:

- Controller is the element that generates the error campaign and computes the obtained results.
- Fault injector oversees the implementation of the fault injection defined by the controller.
- Design under test (DUT) is the target circuit to be studied in the presence of SEUs.
- Monitor is the message passing interface to trace all the system interactions.

A widely accepted classification of the different injection strategies is summarized in next subsections, including a description of each case and some platform examples:

### 3.1. Hardware-Based Fault Injection

Hardware-based fault injection consists in the generation of physical errors into the integrated circuits. The two main options are fault injection with contact and fault injection without contact. In the first category, there are systems like pin-level fault injection, which is based on the idea of perturbing the integrated circuits with faults introduced at the pins that emulate both external and internal faults [21]. Some tools in the literature are: RIFLE [22], FOCUS [23], MESSALINE [24] and AFIT [25]. On the other hand, fault injection without contact is based on the idea that the injector has no direct physical contact with the design under test. In these cases, an external source produces a physical phenomenon such as a heavy ion radiation that interacts with the circuit and produces the faults. Some tools can be found in literature, e.g., FIST [26] and MARS [27].

### 3.2. Software-Based Fault Injection

Software fault injection (SFI) artificially inserts faults and error states into a running software system. These errors can be inserted during compile time or run time.

The ones based on errors during compile time introduce the faults into the source code or the assembly code of the program under test.

In the case of faults inserted during run time, a trigger mechanism is necessary to insert the faults. This trigger is usually generated via:

- A timeout, where a timer expires launching the injection.
- A software trap where the control is transferred to the fault injector module.
- A code insertion alters the program instructions causing the fault injection.

Among others, some related tools are: FERRARI [28], Orchestra [29], FTAPE [30], FIAT [31] and XCEPTION [32].

### 3.3. Simulation-Based Fault Injection

Simulation-based fault injection is a mechanism where the design under test (DUT) is simulated through a Hardware Description Language like VHDL or Verilog and the upsets are injected via software. The main options that can be found in this kind of systems are:

- Those which modify the high-level description of the target design with a saboteur module, which is in charge of the fault injection process.
- Those which use the built-in commands of a simulator like “force”, to inject errors into the simulation of the design, not in the hardware description of the design itself.

As an example, some tools based on these techniques are: SST [33,34], MEFISTO [35] and VERIFY [36]. In all these cases, the failure model described is for an ASIC. This means that SEUs are injected in the memory elements of the design such as flip flops, strictly examining its behavior in the presence of SEUs.

### 3.4. Emulation-Based Fault Injection

Emulation-based fault injection consists in a mechanism that implements the design under test into an FPGA. Unlike the simulation option that uses a circuit high-level description running into a commercial simulator, emulation requires a synthesizable implementation on an FPGA. For these platforms, the development board is connected to a host computer used to: (i) define the fault injection campaign, (ii) control the injection experiments and (iii) display the results.

Some examples of emulation-based fault injection systems for SRAM-based FPGAs are: FT-UNSHADES [37], FLIPPER [38], SPFFI [39], and XRTC [40].

For some of the above solutions, the ASIC failure model (errors into the user memory elements like sequential logic of the target circuits) is not always supported. This is because the vulnerability of SRAM-based FPGAs designs to soft errors is higher than ASIC implementations because of the resources dedicated to memory for programming the board, not for the circuit (device configuration memory). As a rough comparison, the number of the user memory bits can be 10 times higher than RAM bits and 300 times higher than flip-flop bits for an FPGA. SEUs in these configuration bits could cause permanent errors on the FPGA implementation of the design. This fault model is carried out by modifying one bit of the information stored in the configuration memory, also known as bitstream. Each campaign consists in doing a bit flip to each of the bits that belong to the configuration memory, followed by one run-time reconfiguration to restore the original state of the design, avoiding accumulative effects (in the case of SEUs).

## 4. Radiation Effects on SRAM-Based FPGAs

**Figure 5:**
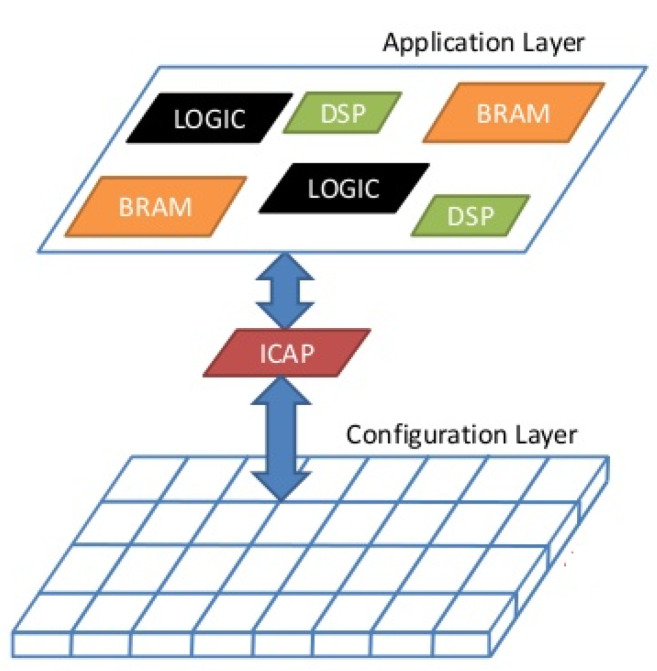
Xilinx FPGA conceptual layers: Application and Configuration layers, extracted from [41].

To understand the effects of radiation on SRAM-based FPGAs, the abstraction of layers in the device is described, as shown in Figure 5. The two main layers that stand out are:

- Application layer: includes the logic and memory elements managed by the user’s design.
- Configuration layer: includes the logic and memory elements that allow the designer to configure the logic and routing resources in the application layer.

SRAM-based FPGAs are sensitive to SEUs. Depending on which layer is struck by particles, the effects can produce different consequences:

- SEUs induced in the Application layer are shown as transient errors that could alter the stored data or the state of the user logic memory elements such as Flip-Flops or BRAMs (ASIC failure model).
- SEUs affecting the Configuration layer produce persistent errors that can be reverted using a reconfiguration process. This kind of error consists in a bit flip which, in case of being an essential bit, may change the design functionality. This can have two consequences:
  ▪ Change a routing element connection or disconnecting internal wires.
  ▪ Change a logic element modifying the behavior of a LUT belonging in a CLB.

SEUs in the configuration layer are the most common type of errors in SRAM-based FPGAs because a high percentage of all the memory elements in the device are SRAM cells [42]. A summary of SEU consequences is presented in Table 1.

## 5. Fault-Tolerant Techniques for FPGAs Based on Reconfiguration

This section is focused on SEUs affecting the configuration layer, as they are more likely to occur. To overcome their effects, some techniques that exploit the particular reconfigurable capabilities of the FPGAs to detect and correct persistent errors in the configuration memory are detailed next:

- Scrubbing is a technique used to correct and prevent errors in the information stored in memory. In FPGAs, scrubbing can be used to mitigate both persistent errors in SRAM cells (i.e., the configuration memory) and transient errors in user-memory elements such as BRAMs. To perform configuration memory scrubbing, the configuration memory data must be read sequentially from the start to the end and compared to the original configuration bitstream or an error check code such as a cyclic redundancy check (CRC) [43].
- Dynamic partial reconfiguration allows run-time reconfiguration without application layer interruption. This technique cannot detect errors by itself, so it must be combined with other error detection techniques such as those based on redundancy. These correction techniques take advantage of the subdivision of the configuration memory into frames, which contain information related to the configuration of specific parts of the design.

All the features presented in the previous sections are useful to understand the principles that drive an FPGA reliability analysis and the related fault injection tools for FPGA designers.

## 6. An Emulation Framework for Fault Injection

At this point, a step by step tutorial that describes the fault injection tool based on emulation is presented. The setup is based on Xilinx technology. The main modules that make up the system are the following:

- The LogiCORE IP Soft Error Mitigation (SEM) Controller version 4.1 [44].
- A Nexys 4 DDR board based on the latest Artix-7™ FPGA from Xilinx [45].
- A design under test (DUT) implemented for FPGA, in order to measure its dependability in case of soft errors.
- A universal asynchronous receiver transmitter (UART) module to implement communication between both FPGA and host.

An overview of the whole system is explained next.

### 6.1. Soft Error Mitigation (SEM) IP Controller

SEM IP core is the fault injection engine for the emulation process. This IP core, supported by Xilinx, is a solution to detect and correct soft errors in the Configuration Memory of Xilinx FPGAs.

This module can read the configuration memory to look for errors. In case of discovering any altered bit, it is flipped by the SEM IP to correct it. This ability to read and write into the configuration memory is used to manage the fault injection in a non-invasive way. The manager based on SEM IP could inject bit flips into the configuration memory, testing the reliability of the solution implemented for an FPGA after the injection. SEM IP controller is employed because it is the only tool in Xilinx FPGAs that allows checking the status of the Configuration Memory of the devices to look for faults. Customized controllers were not considered as SEM IP was already designed, validated and tested by the manufacturer of the boards.

**Figure 6:**
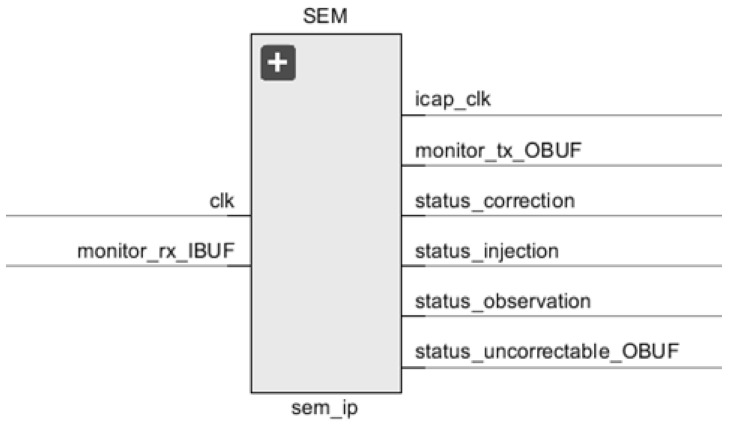
SEM IP entity.

In order to add the SEM IP core to a Vivado project, as it can be seen in Figure 6, it requires to be instantiated from the IP catalog.

To do so:

- The clock input signal (clk) should be mapped to the global clock of the design.
- The Monitor Interface is an UART that serializes status information generated by the SEM IP controller for serial transmission between the host computer and the FPGA over the TX/RX lines:
  ▪ Monitor RX signal receives as inputs the commands that SEM IP core interprets in order to perform functions like, for example, injections.
  ▪ Monitor TX signal reports from the SEM IP core, the new states achieved among these: idle, initialization, observation, injection, correction, fatal error and classification.

Other signals will be described in later sections. Finally, it is important to remark that the FPGA families supported by the SEM IP core are the Zynq-7000 all Programmable SoC and the 7 Series.

### 6.2. Nexys 4 DDR Based on Artix-7 FPGA

Among all the compatible FPGAs, the supported device chosen for this tutorial has been the Nexys 4 DDR based on an Artix-7 (15,850 logic slices, consisting of four 6-input LUTs and 8 flip-flops).

For the implementation of the fault injection platform based on the SEM IP core presented in this tutorial, the next resources are used to automatize the injection functionality and its monitoring services:

- Peripheral module (Pmod) ports, for the serial communication tasks between the FPGA and the computer, supporting monitoring functions like send and receive data. The Pmod is an input/output interface board, developed by Digilent, that enables a simple connection between the FPGA and other standardized sockets from other external devices or even computers. Pmod ports avoid welding wires to the FPGA as they are convenient and easy to plug modules.
- FPGA configuration reset button, allows to reset the FPGA after each fault injection. Note that because of an emulated SEU which permanently alters the configuration layer, the FPGA requires to be reset. After these reset conditions, the FPGA must be configured again loading the original bitstream. For this purpose, a flash memory included in the board contains a copy of the stored design, in order to automatize the configuration after each fault is injected, avoiding a manually load through the Vivado tool.

### 6.3. Design for the Experimental Set-Up

**Figure 7:**
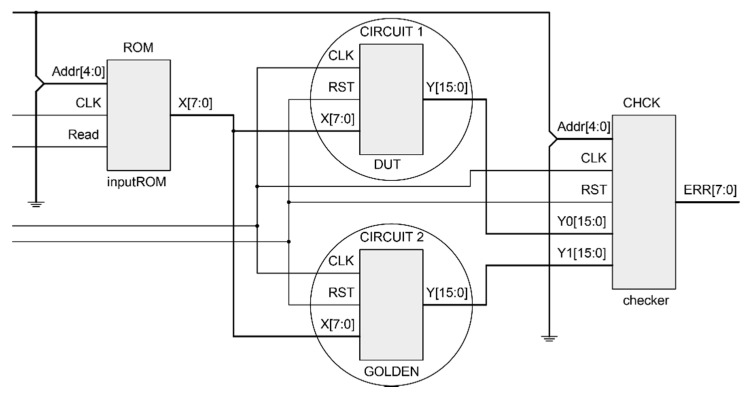
Design for the experimental setup.

The proposed structure for the experimental set-up in this tutorial is shown in Figure 7. It consists of: a ROM, where the input stimuli are contained; a twin circuit CIRCUIT 1 and CIRCUIT 2 with the original behavior, and a checker module (CHCK) that performs a comparison between the two copies in order to detect if any error happened. Only CIRCUIT 1 is considered as the DUT where errors will be injected by the SEM IP controller, and CIRCUIT 2 keeps the original behavior and acts as a golden copy that, by means of a comparator (CHCK), validates if the outputs from CIRCUIT 1 after injection match the expected values.

The same validation could also be done by producing a pre-processed output file free of errors (with the expected outputs). The output of the DUT would then be compared to the output file for the same circuit in presence of SEUs. After that, a data post-processing in the host PC calculates the number of errors detected.

In http://www.nebrija.es/aries/acme.htm an online copy of a real project based on an FIR filter structure can be found with a brief document that includes all the process, step by step. This example can be used as a case study to make a full demo of the fault injection process.

### 6.4. Universal Asynchronous Receiver-Transmitter

**Figure 8:**
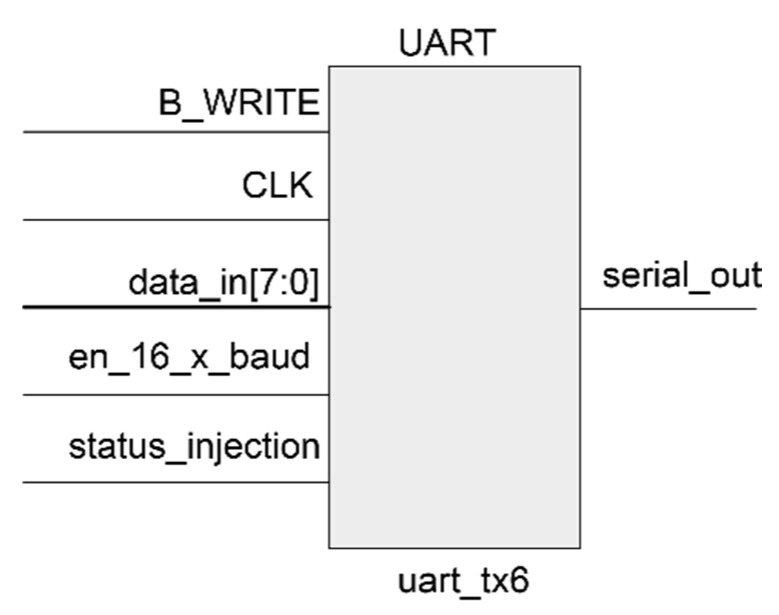
UART model.

The UART module is included as another part of the design to manage serial communications between the host computer and the FPGA over the serial_out line as is shown in Figure 8.

**Figure 9:**
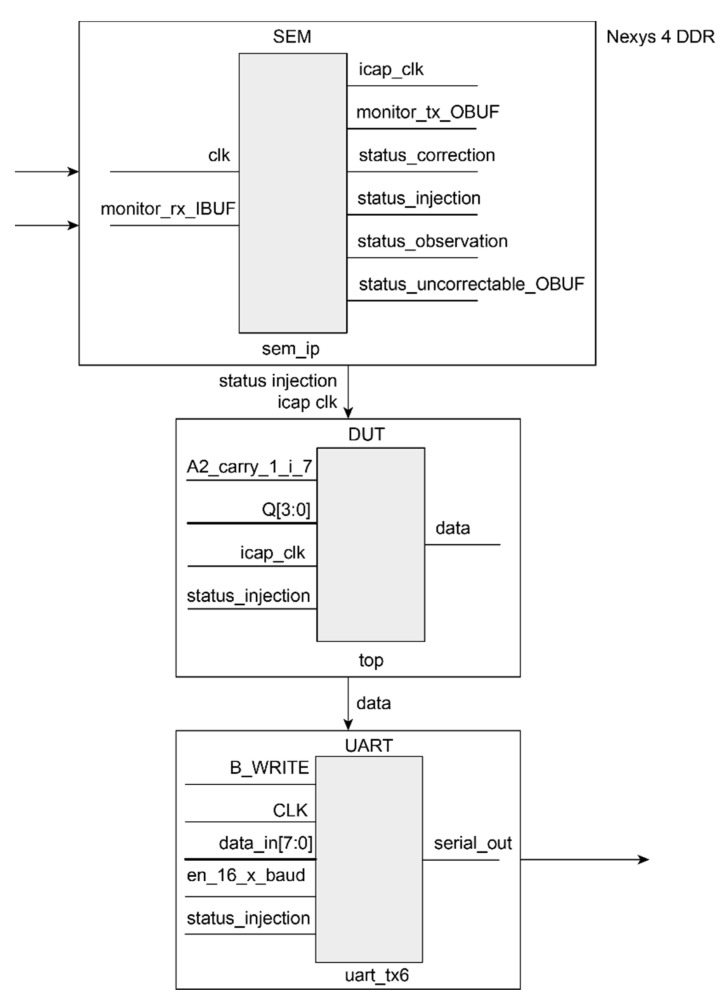
Source files overview.

This UART allows a monitor process similar to the one supported by the SEM IP controller, but in this case, it is used to send to the host computer if there was an error in the output of the system after each injected SEU. In order to connect this UART model to the host computer, another USB to UART converter such as a Pmod USBUART has been used, and the mapping must be included in the constraint file again. As a summary (Figure 9), the design implemented in the Nexys 4 DDR board is composed of:

- DUT composed of ROM, CIRCUIT 1, CIRCUIT 2 and the CHCK.
- UART for communication of the errors.
- SEM IP core included in Vivado.

## 7. Emulation Workflow Step by Step

Next, the different steps required to implement fault injection emulation based on the architecture defined in the previous section will be described. In this section it will be shown that two projects will be necessary. The first one is the main workspace, where the design for the experimental set-up, the UART and the SEM IP controller will be integrated for the target reliability tests. The second one is only necessary to extract and reutilize the SEM IP core files into the first one and it will be removed after this task is completed.

Now, a step by step description of the emulation is detailed. First, a new RTL project for Vivado (the first one), is created including the HDL (Table 2) files that refer to both the design for the experimental set-up and UART modules. The inclusion of the SEM IP controller will be shown in detail later.

**Figure 10:**
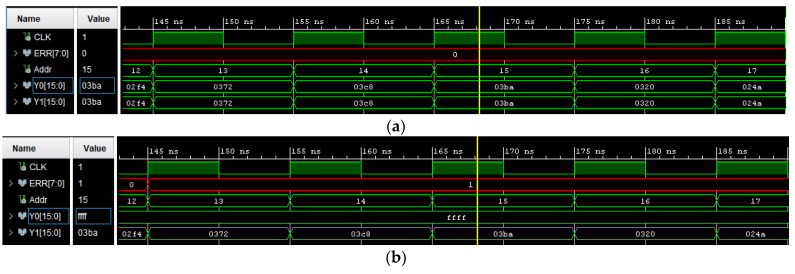
(**a**) Simulation without an SEU. (**b**) Simulation with an SEU.

Also, at this point the board model has to be selected. For this tutorial, the board is a Nexys 4 DDR with Artix-7 FPGA. Once the DUT, the golden copy and the checker are implemented, and before continuing with the process, a good practice would be to simulate and check the design for the experimental set-up. For instance, Figure 10a, the CHCK output is included for a sample circuit with no injected errors. In this trivial example both outputs defined as Y0 (CIRCUIT 1) and Y1 (CIRCUIT 2) are equal in the whole simulation, and the checker output (ERR) takes a value of 0, which indicates that no errors occur.

On the contrary, the next simulation (Figure 10b) shows an error detected by the checker due to an SEU inserted via force command in the DUT. It can be observed that both outputs are different (Y0 has a value of ffff and Y1 has the correct value of 03ba) and the checker detects the error changing its output, ERR (at 168 ns), with a code different from the right one.

After the verification of the experimental environment, the SEM IP controller is required to be instantiated in order to enable and control the fault injection process. This core can be found in the Vivado IP Catalog, and its maximum clock frequency meets the frequency of the board (for Nexys 4 the threshold is 100 MHz). Below this frequency the SEM IP controller works properly. Also, the controller options must be enabled.

**Figure 11:**
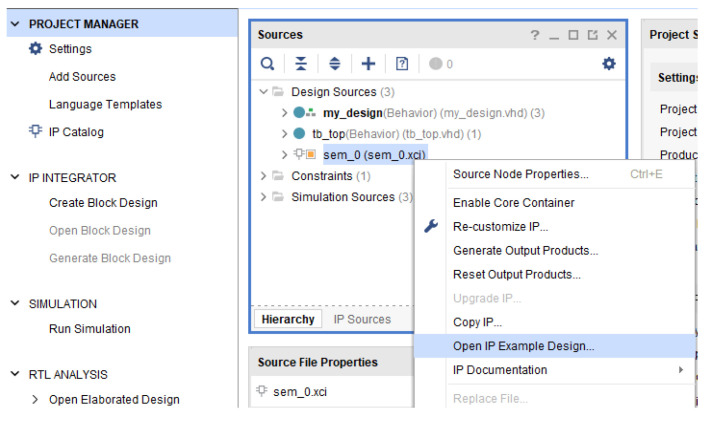
Generation of the SEM IP files through IP example.

**Figure 12:**
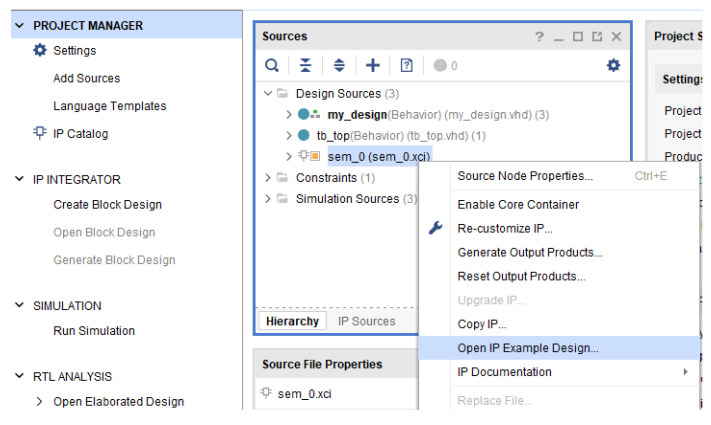
SEM example supported by Xilinx.

**Figure 13:**
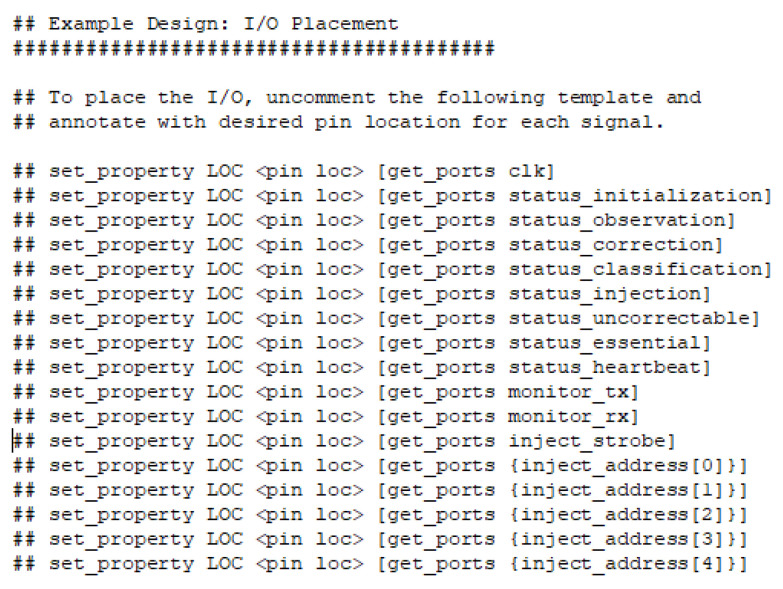
Original SEM IP constraint file.

At this point, a new Vivado project (the second one), will be opened with the purpose to extract the SEM IP source files and integrate them into the main project in order to complete the design for the experimental set-up (Figure 11). This is an example project included in the SEM IP distribution. Next, the newly generated project source files must be copied to the original project including the constraint file (.xdc), generated in the example itself (Figure 12 and Figure 13). This constraint file extracted from the example includes a valid initial mapping for the SEM IP core interface with the board. Both, required source and constraint files, are allocated into the imports folder of the “sem_0_ex” project:

*“sem_0_sem_cfg, sem_0_sem_example, sem_0_sem_mon, sem_0_sem_mon_fifo, sem_0_sem_mon_piso, sem_0_sem_mon_sipo and “sem_0_sem_example.xcd”.*

**Figure 14:**
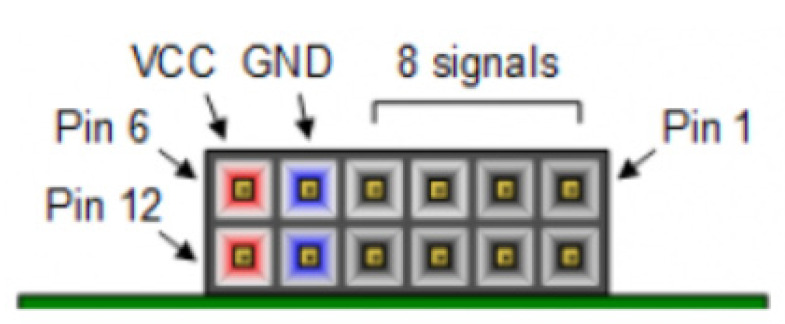
Pmod connectors. Front view.

**Figure 15:**
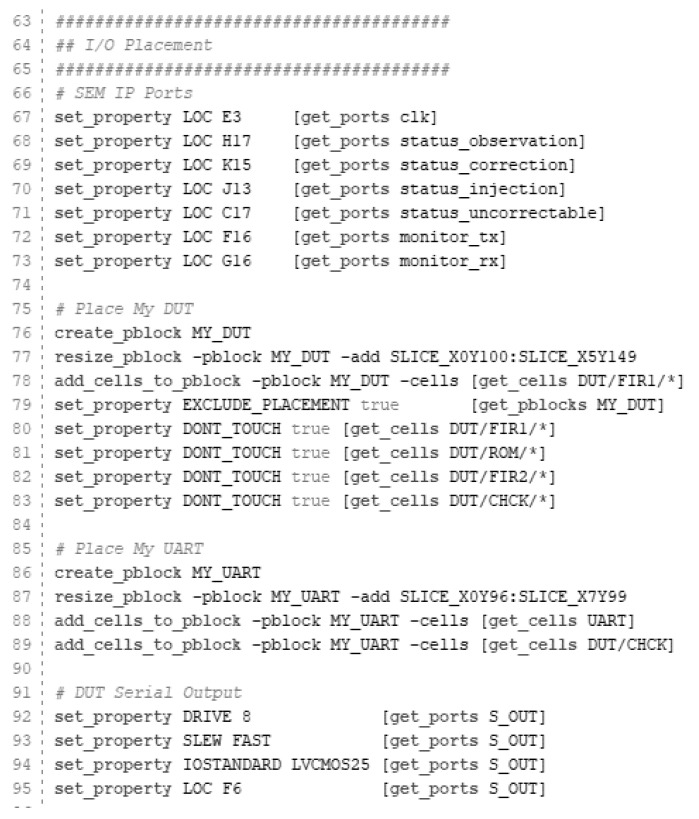
Constraint file example.

Before the synthesis and implementation processes, the original constraint file (obtained from the SEM example directly) can be customized. Pmod connectors are described for the Nexys 4 in Figure 14 and Table 3, while Figure 15 shows one of the possible configurations for this example.

For this tutorial, Pmod JB and Pmod JC have been selected for both SEM IP monitor interface (RX/TX) and UART (S_OUT) respectively. It is important to highlight that this configuration is just an example, any other pin of the Pmod can be assigned to the serial interface without any modification in the final behavior, just two ports need to be available.

The Nexys 4 board includes a single 100 MHz crystal oscillator connected to pin E3, which can be used as a master clock for the system. Other examples are both monitor_tx and monitor_rx lines (SEM IP monitor interface or UART) configured in the G16 and F16 belonging to the Pmod JB. Pin C17 is hard-wired and used to send a reset signal to the FPGA configuration reset button when the SEM IP cannot recover the original design after a failure provoked by an SEU. Finally, the DUT serial output that indicates if an error is provoked or not, is registered in the F6 pin.

**Figure 16:**
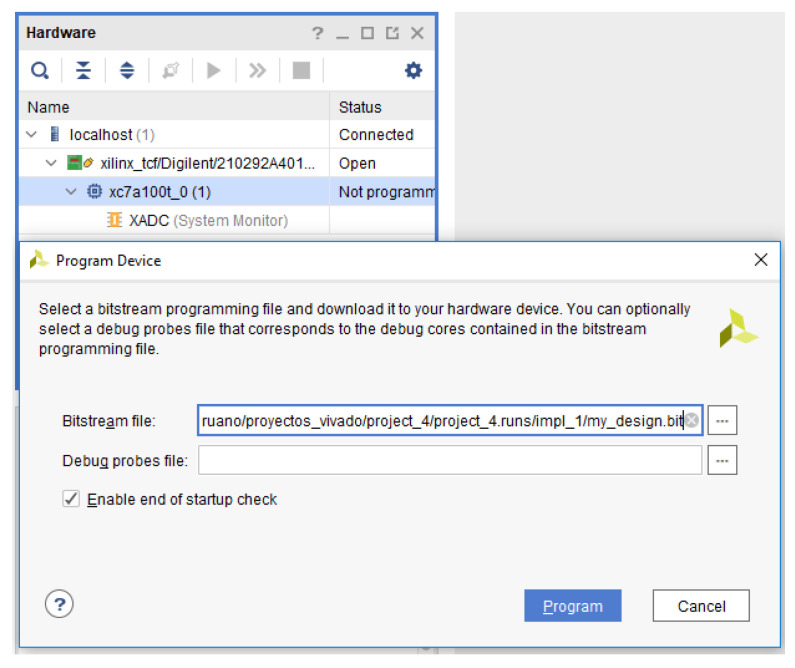
Program SRAM configuration memory.

At this point, everything is ready to implement the design into the FPGA. For this purpose, the bitstream can be generated in order to program the FPGA. For it, the hardware manager must be opened in order to auto-detect and program the device as shown in Figure 16.

**Figure 17:**
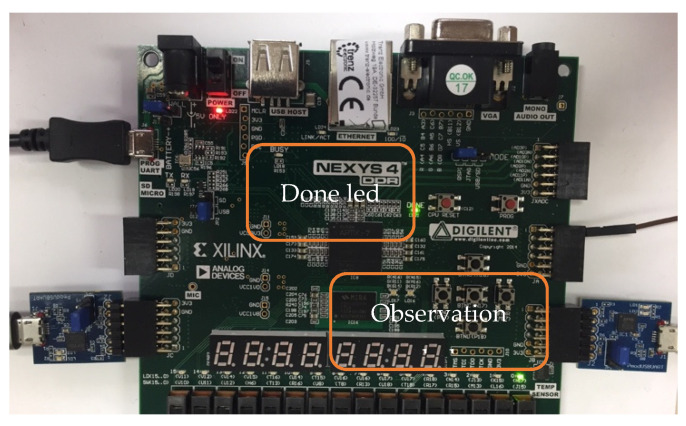
Device programmed with SEM IP observation mode (H17 led).

Once the device has been loaded with the resultant bitstream, the appearance of the board is the one included in Figure 17. As it can be noticed, both green LEDs are lit. The first one is the “FPGA programming done” LED and the second is defined in the constraint file as an SEM IP signal denoting that the module is in observation mode. Therefore, it indicates that the SEM IP is ready to operate.

**Figure 18:**
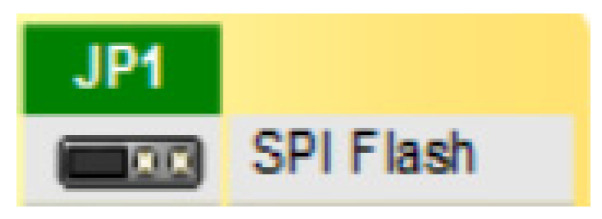
Jumper for SPI Quad mode Flash programming mode.

Another SEM IP output mapped through the constraint file is the status uncorrectable in the pin C17. A bridge between this pin and the FPGA configuration reset button is done, for auto-reconfiguration purposes. This reset works with negative logic, so it requires to be inverted in the original sem_0_sem_example file:

status_uncorrectable ≤ not (status_uncorrectable_internal);

Another important point is that, since the Artix 7 FPGA is based on SRAM volatile memory, it relies on the integrated Quad-SPI flash memory to store the configuration between power cycles. When this flash device has been programmed, it can automatically reconfigure the FPGA at a subsequent power-on or reset event as determined by the mode JP1 jumper setting (Figure 18). Regardless of which board is going to be used, it is recommended to include a flash memory because of the constant need for reconfiguration, in order to automatize the process.

**Figure 19:**
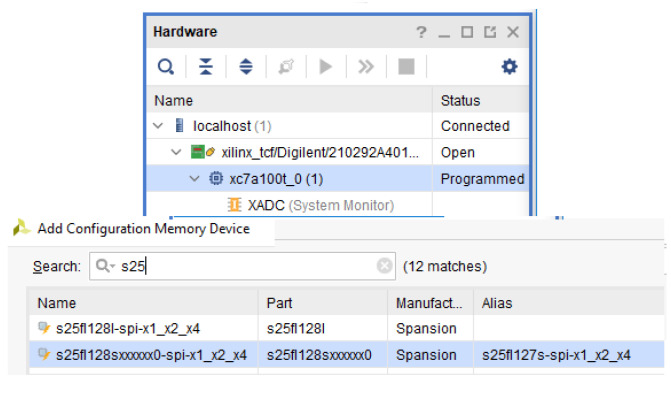
Flash memory device for Nexys 4 DDR.

As can be noticed in Figure 19, in order to use it, this memory has to be added through the Vivado hardware manager and it allows the FPGA configuration memory to be auto-reprogrammed from the Quad-SPI Flash (Spansion part number S25FL128S) previously configured with the original design.

**Figure 20:**
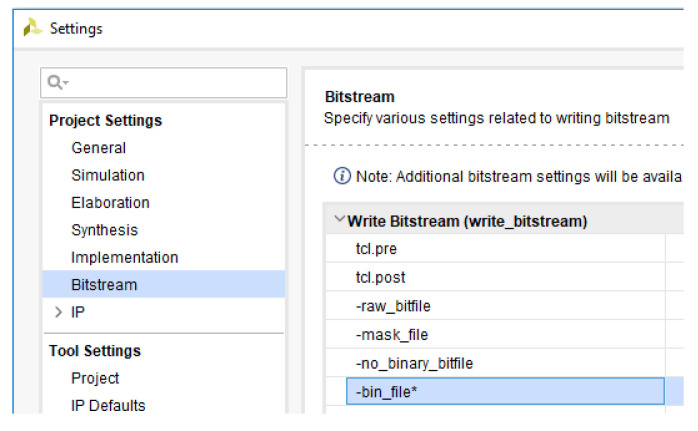
File generation for flash memory (.bin).

**Figure 21:**
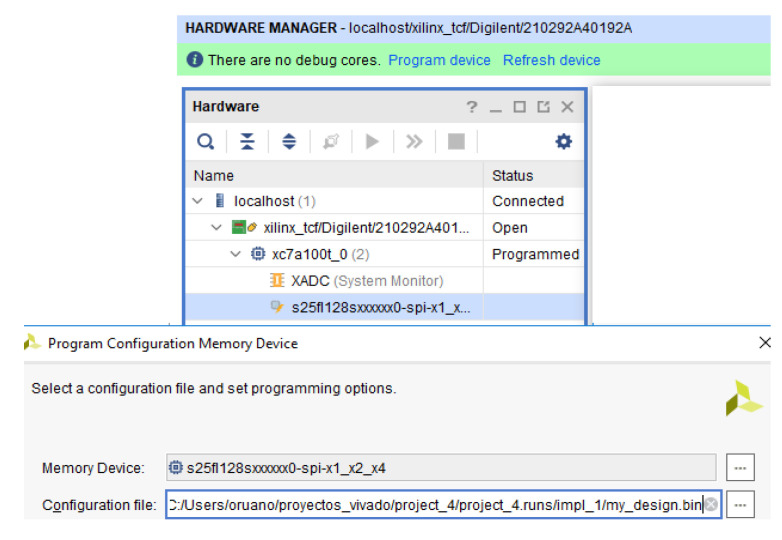
Flash memory load.

This original design needs to be saved in a.bin file as shown in both Figure 20 and Figure 21.

It should be mentioned that the next couple of lines must be included in the constraint file to enable the possibility to load the original bitstream from the flash memory:

set_property BITSTREAM.CONFIG.SPI_BUSWIDTH 4 [current_design]

set_property CONFIG_MODE SPIx4 [current_design]

Once the manager has been configured and before proceeding with the interactive process, let us describe the different states that can be adopted by the SEM IP.

**Figure 22:**
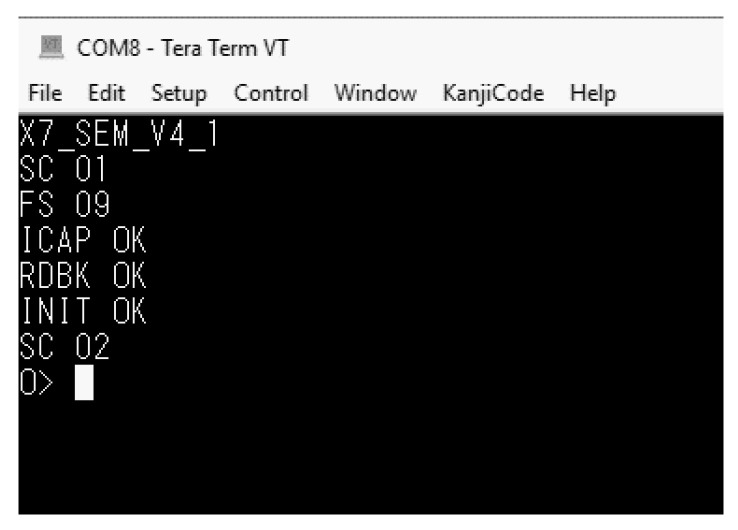
SEM IP Controller initialized. Ready to receive commands for the injection process.

**Figure 23:**
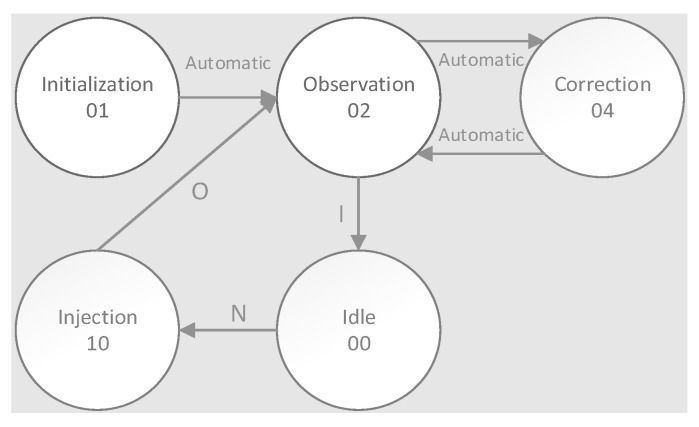
SEM IP state diagram.

- *Initialization (01)*: Once the configuration has been completed, the FPGA sends the global set/reset signal and the SEM IP controller starts. If the initialization process has been completed in the correct way, the controller moves to the observation state showing in the monitor the next (Figure 22):

- *Observation (02)*: When the controller is in the observation state, status_observation variable is set, and the SEM IP controller watches the FPGA configuration looking for errors. In case of an error, the controller transits to the correction state to recover the original configuration automatically. If no error exists, when the SEM IP controller receives a command, it is executed. Both “enter idle” (moves to idle state) and “status report” commands are supported in the current state.
- *Correction (04)*: When the controller is in the correction state, status_correction variable is set. If the SEM IP is setup for correction to repair or correction by enhanced repair, it tries to correct the error through algorithmic methods. If the error can be corrected, the SEM IP instance uses the partial reconfiguration feature in order to modify the affected frame with the good information and resets the status_uncorrectable variable. In case that the error cannot be corrected, the controller sets the status_uncorrectable variable. When this situation occurs, the FPGA must be reconfigured. Once correction is completed, the controller moves back to observation state.
- *Idle (00)*: When the controller reaches this state, it is prepared to execute both error injection and software reset commands which are supported in this state.
- *Injection (10)*: When the controller is in this state, the injections action can be performed. It happens when an error injection command is executed from the previous idle state. The emulation of the strike of one SEU into the configuration memory is achieved by flipping the bit which corresponds to the memory address provided in the error injection command. After each injection, the controller moves from the injection to the idle state automatically. At the end of error injection, the controller transits to the observation state (Figure 23).

After the SEM IP states have been presented, we are ready to interact with the fault injector manager through the enabled communication ports defined in the constraint file. As it was said in a previous section, connecting a serial peripheral module such as a Pmod USBUART converter to the FPGA port, where both “monitor_tx” (G16) and “monitor_rx” (F16) signals has been mapped from the constraints file (in this case Pmod JA), will allow to send and receive commands from the SEM IP core. In order to undertake this communication, a serial terminal program e.g., “Tera Term” in this tutorial, is used like a monitor interface to register any interaction. As it was mentioned in the SEM IP controller section, the monitor interface (SEM IP UART included) consists, among others, of two signals implementing an RS-232 compatible protocol, for a full duplex channel to exchange commands and status. The next configuration is used for a Tera Term terminal:

Band: 9600
Settings: 8-N-1
Flow Control: None
Terminal Setup: VT100
TX Newline: CR (Terminal transmits CR [0x0D] as end of line)
RX Newline: CR+LF (Terminal receives CR [0x0D] as end of line, and expands to CR+LF [0x0D, 0x0A])
Local Echo: NO

To configure the communication bit rate, the parameter V_ENABLETIME declared in the sem_0_sem_mon, which is a MON Shim implementation for communication with external RS232 devices, must be set according the following Equation (1):

(1) $$V_{ENABLETIME}=round\;to\;integer\;\left[\frac{input\;clock\;frecuency}{16*nominal\;bitrate}\right]-1$$

For example, for a baud equal to 9600, the V_ENABLETIME will be 650. If the baud requires to be increase to 230,400, then the V_ENABLETIME will be 26. The command set available from the Error Injection Interface are the following (case sensitive: uppercase):

- command sends the controller to the observation state.
- **I** command sends the controller to the idle state.
- **S** command requests a status report.
- **N** command performs an error injection. This command is only supported in the idle state. The interface is: N {10-digit hex value}
- **R** command performs a software reset. This command is only supported in the idle state. The interface is: R {2-digit hex value}

All this information is enough to generate the fault injection campaigns for each configuration bit. Obviously, at the same time that commands are sent to the SEM IP controller via Pmod1 (JB), the output of the DUT will be received through the Pmod2 (JC) and the number of errors that provoke failures in the output will be counted.

Next section deals with the performance problem for the emulation in SRAM-based FPGAs. If the campaign is exhaustive around the whole configuration memory, then the time required to run a fault injection could be excessive.

## 8. ACME: Speeding Up the Injection Performance

Essential bits defined by Xilinx programs are related to the whole system, including the SEM IP, the ROM, the DUT, the golden copy and the checker (Figure 7). As the objective is to inject in the DUT to validate its fault tolerance against SEUs in the configuration memory, the injection process must be driven selecting only those bits from the configuration memory that may have impact on the behavior of the design under test. It is an attempt to optimize the campaign performance eliminating injections at insignificant areas.

**Figure 24:**
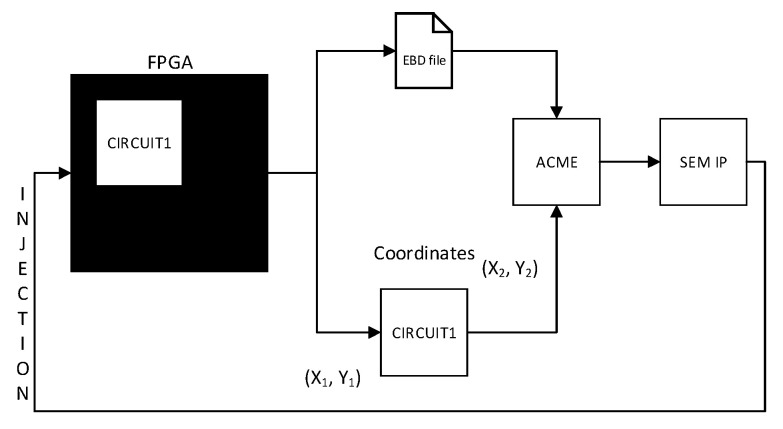
Integration of the ACME tool into fault injection process.

In order to carry out a driven fault injection campaign that can be able to improve the time emulation, a tool called ACME [13] is used. ACME is an open-source tool designed to translate the configuration memory essential bits of a Xilinx SRAM-based FPGA region into injection addresses for the Xilinx SEM IP controller. ACME needs the EBD file of the complete design together with the pBlock coordinates of the design under test as inputs to generate a text file containing the injection addresses of the specified range as is shown in the Figure 24.

**Figure 25:**
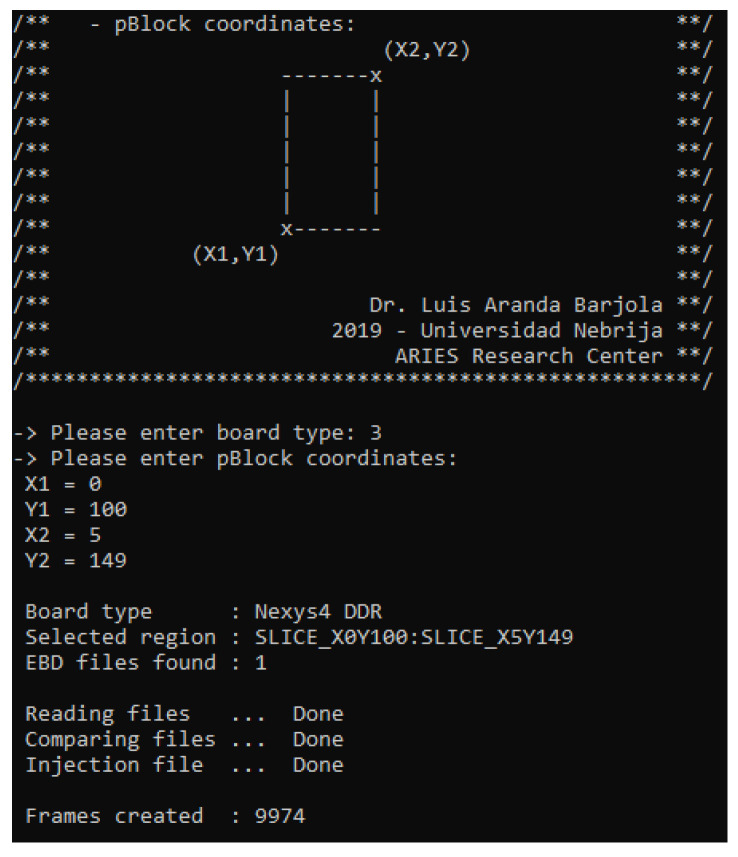
Interface ACME tool.

ACME currently supports the ZedBoard, the Basys3 Artix-7, the Nexys 4 DDR, the ZC706, and the KCU105 UltraScale, but it can be easily configured to work with other boards. When ACME is started (Figure 25), the required data are the following:

- Model of board (Nexys 4 DDR for this tutorial).
- An EBD file that lists all the essential bits in the bitstream.
- Coordinates of the pBlock where the DUT is allocated into the FPGA. This information might be defined in the constraint file, through the routine:

*resize_pblock -pblock MY_DUT -add SLICE_X0Y100:SLICE_X5Y149*

ACME computes these inputs generating as a result, a text file named FrameRange. It collects a subset of essential bits belonging to the pBlock area. For instance, in the above Figure 9974 essential bits are identified from the .ebd file. These bits are translated by ACME into configuration memory addresses that will be used by the SEM IP controller to conduct one injection per address, in the following way:

*N {10-digit hex value}: N C00XXXXXXX*

As it can be noticed, this routine is composed of the N injection command and the address translated by ACME that corresponds with an essential bit belonging to the pBlock. More details about ACME tool, can be found in the web site of the ARIES research group [46].

## 9. Automating the Fault Injection Process

**Figure 26:**
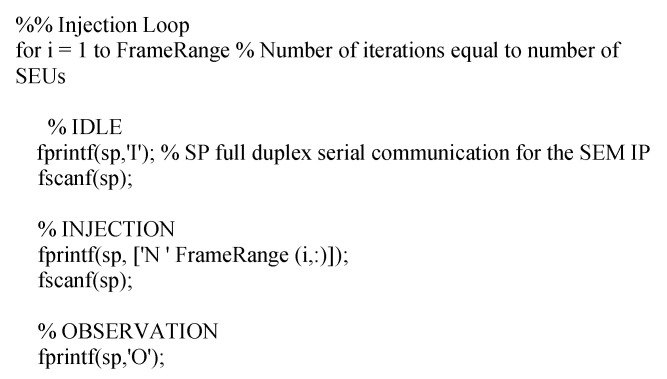
Example of fault injection loop in MatLab.

In order to automate the fault injection process, a Matlab script is used for this purpose (Figure 26). The input for the script is the FrameRange file, with all the information about sensitive bits in terms of configuration memory addresses. Each one of the injections (one per address) are sent to the SEM IP controller through the monitor interface as is showed.

At the same time, through the Pmod 2 (JC) the output of the DUT (S_OUT), would be received to compute the number of errors produced during the fault injection experiment.

As a summary of this tutorial, a simple case of study based on a FIR filter structure is provided online with direct links to the ACME tool; an online copy of a full project; and a step-by-step documentation on how to create a workflow including all the screenshots and code for the different development kits involved: http://www.nebrija.es/aries/acme.htm

## 10. Conclusions

In this tutorial, a complete revision of an emulation-based fault injection platform for SRAM-based FPGAs has been conducted to evaluate the behavior of electronic components that are required to work in the presence of radiation. To illustrate the tutorial, the FIR filter of a superheterodyne receiver has been selected as an example of a communication system that can have malfunctions due to the effects of radiation in the sensitive elements of the FPGA. The tutorial presents an affordable and efficient workflow based on the Xilinx technology together with the required tools to analyze the effects of the faults caused by radiation. The aim of this tutorial is to help hardware developers to test the reliability aspects of a design in a straightforward and comprehensive way. With this in mind, the integration of the ACME tool in the workflow and its relationship with the Xilinx Soft Error Mitigation controller are also detailed to improve the reliability results obtained and to reduce the execution time required to perform the fault injection campaigns.

## Figures and Tables

**Table 1:** Consequences in SRAM-based FPGAs [42].

Layer		Element	SEU Consequence
Configuration Layer	Routing	Muxes	Wrong input selection, open net, wrongly driven or left open
		PIP	Wrong connection o disconnection between nets
		Buffers	Output net wrongly driven or left open
	Logic	LUT	Wrong function inputs and outputs
		Control bits	Wrong function inputs and outputs
		Tie Offs	Wrong function initialization
Application layer	RAM Blocks		Wrong application data
	CLB Flip-flops		Wrong application data or state

**Table 2:** Projects files before SEM IP core.

Modules	Files
Design for the experimental set-up	rom.vhd
	circuit1.vhd
	circuit 2.vhd
	checker.vhd
UART	uart.vhd

**Table 3:** Nexys4 DDR Pmod pin assignments.

Pmod JA	Pmod JB	Pmod JC	Pmod JD	Pmod JXDAC
JA1: C17	JB1: D14	JC1: K1	JD1: H4	JXADC1: A13 (AD3P)
JA2: D18	JB2: F16	JC2: F6	JD2: H1	JXADC2: A15 (AD10P)
JA3: E18	JB3: G16	JC3: F2	JD3: G1	JXADC3: B16 (AD2P)
JA4: G17	JB4: H14	JC4: G6	JD4: G3	JXADC4: B18 (AD11P)
JA7: D17	JB7: E16	JC7: E7	JD7: H2	JXADC7: A14 (AD3N)
JA8: E17	JB8: F13	JC8: J3	JD8: G4	JXADC8: A16 (AD10N)
JA9: F18	JB9: G13	JC9: J4	JD9: G2	JXADC9: B17 (AD2N)
JA10: G18	JB10: H16	JC10: E6	JD10: F3	JXADC10: A18 (AD11N)

## Data Availability

http://www.nebrija.es/aries/acme.htm.
